# Research on the Directional Measurement Method of Three-Dimensional Electric Field Intensity Components of the Atmosphere Based on the Geographic Coordinate System in the Airborne Model

**DOI:** 10.3390/s25175595

**Published:** 2025-09-08

**Authors:** Wei Zhao, Zhizhong Li, Haitao Zhang

**Affiliations:** 1 School of Intelligent Manufacturing, Huzhou College, Huzhou 313000, China; zhaowei@zjhzu.edu.cn; 2 State Key Laboratory for Disaster Prevention & Mitigation of Explosion & Impact, Army Engineering University of PLA, Nanjing 210007, China

**Keywords:** coaxial error, electric field sensor, modified model, tansig, particle swarm optimization

## Abstract

**Highlights:**

**What are the main findings?**

- Modified model for three-dimensional electric field orientation decomposition based on geographic coordinates.
- Nonlinear dynamic inertia weight adaptive particle swarm optimization algorithm based on the tansig function.

**What is the implication of the main finding?**

- The three-dimensional electric field directional decomposition model based on geographic coordinates has been optimized.
- The measurement accuracy of the atmospheric electric field intensity components based on the geographic coordinate system was improved.
- A three-dimensional electric field orientation measurement system for unmanned aerial vehicle (UAV) airborne mode was designed, and a more accurate mathematical model for achieving high-precision orientation measurement of three-dimensional electric field intensity in the air based on the geographic coordinate system is provided.

**Abstract:**

Due to the difficulty of achieving absolutely precise coaxial installation of measurement components, a coaxial error will occur between the measurement axis of the sensor and that of the electronic compass when conducting the measurement of the airborne atmospheric electric field intensity based on the geographic coordinate system under the airborne mode of unmanned aerial vehicles. This error leads to less satisfactory measurement accuracy of the atmospheric electric field intensity components based on the geographic coordinate system. In this study, the angle between the measurement axis of the sensor and that of the compass is set as the parameter to be identified, and a modified model for three-dimensional electric field orientation decomposition based on geographic coordinates is constructed. In view of the characteristics of the nonlinear equations of this model, an algorithm based on tansig–Nonlinear Dynamic Inertia Weight Adaptive Particle Swarm Optimization (NDIWAPSO) is proposed to solve the modified model, successfully addressing the problem of insufficient measurement accuracy of the electric field intensity components in the geographic coordinate system caused by the coaxial error. The experimental results show that the parameters of the three-dimensional electric field orientation decomposition modified model can be accurately identified by the algorithm proposed in this paper, improving the measurement accuracy of the atmospheric electric field intensity components based on the geographic coordinate system and laying a necessary foundation for lightning warning.

## 1. Introduction

Lightning can easily have a serious impact on people’s lives and property safety [1] and has received widespread attention due to its frequent occurrence [2]. The occurrence of lightning is sudden, which makes the work of lightning forecast and early warning rather difficult [3,4,5]. The use of atmospheric electric field meters, lightning location, radar and other means to carry out lightning monitoring and early warning is one of the important measures to prevent and reduce the losses caused by lightning disasters [6,7], The atmospheric electric field meter is capable of measuring the changes in the ground electric field caused by the lightning process, directly reflecting the charge state of thunderstorm clouds and holding a significant position in lightning early warning [8,9].

The electric field sensor, as the core component of the atmospheric electric field meter, has been studied from two aspects: the sensor principle and structure. Specifically, the field-milling type [10,11] and those based on Micro-Electromechanical Systems (MEMS) technology [12,13] have been explored. At present, the ground atmospheric electric field detection technology is relatively mature. However, lightning warning requires data on atmospheric electric fields in the air—the airborne electric field detection data can better reflect the distribution of the atmospheric electric field in the air. An atmospheric electric field measurement system based on a field-grinding three-dimensional electric field sensor was studied in reference [14]. This system integrates functions such as electric field intensity signal processing, Global Position System (GPS) positioning, and electric field intensity direction positioning, and can be widely used for atmospheric electric field detection. This system consists of modules such as constant electric field intensity signal processing, GPS positioning, and electronic compass direction identification. Among them, the three-dimensional electric field signal processing system includes sub-modules such as multi-level amplification, filtering, and A/D conversion of electric field intensity signals. Hence, the study of airborne atmospheric electric field detection technology is conducive to accurately obtaining the data of the atmospheric electric field [15].

To conduct directional measurement of the electric field in the air using a three-dimensional (3D) electric field sensor, it is necessary to carry the device on a platform such as a hot air balloon or unmanned aerial vehicle (UAV) [16]. However, the electric field data measured by sensor is the data under its local coordinates; the position, posture and direction of the sensor will constantly change along with the movement of the carrier, resulting in the failure to measure the electric field intensity in the east–west, north–south and plumb line directions, and the lightning location cannot be realized. Aiming at the above problems, Lin studied a directional measurement system that can measure the components of the atmospheric electric field along the geographic north–south, east–west, and plumb directions [17]. Considering the coaxial error between the sensor and the electronic compass, a 3D direction decomposition modified model was proposed [18]; the measurement accuracy of the electric field component has been improved. The Angle error was taken into account, but due to the insufficient calculated accuracy of the Angle, the accuracy of the modified model was insufficient, resulting in a large measurement error. The modified directional decomposition model for the 3D electric field is a nonlinear equation, which is set for the Angle between the measuring axis of the electronic compass and the vertical direction of the sensor’s three groups of induction electrodes. Accurate identification of these Angles is the key to improving the accuracy of the model. For the solution of nonlinear equations, the swarm intelligence algorithm is usually adopted, such as the differential evolution algorithm [19], firefly algorithm [20], and cuckoo search algorithm [21,22], the precision of solving nonlinear equations is improved to a certain extent.

The particle swarm optimization algorithm (PSO) is widely used in solving nonlinear equations, which can be used to optimize the modified model of 3D electric field directional decomposition. Since the convergence accuracy and convergence speed of the PSO algorithm are important indicators for evaluating the algorithm, in order to obtain the global optimal solution, many scholars have further improved the convergence of the algorithm by dynamically adjusting the inertia weight of particles, maintaining the particle diversity mechanism, and increasing the number of independent running times of the algorithm. This paper intends to improve the convergence of the algorithm by dynamically adjusting the inertia weight of particles and ensuring the convergence of the algorithm through multiple independent runs. Many methods of optimizing the algorithm by changing the inertia weight were proposed, such as linear decrease [23], trigonometric function type decrease [24], exponential decrease [25], etc. The optimization ability, to a certain extent, has been improved and falling into the local optimal solution prematurely has been avoided. However, the above algorithms cannot effectively reflect the complex nonlinear behavior in the particle swarm search process, and the convergence rate and accuracy of the algorithm are still insufficient. As a result, the Angle between the sensor and the electronic compass cannot be accurately identified, and the accuracy of the modified model is still insufficient.

In the optimization process of the PSO algorithm, the inertia weight directly affects the algorithm’s optimization ability. When the inertia weight shows a concave function decreasing, the local search ability of the algorithm is superior to the global search ability; it is easy to fall into a local optimal solution, that is, to experience premature maturity. The convex function is just the opposite, which is prone to cause the convergence speed of the algorithm in the later stage of iteration to slow down. Taking into account the optimization ability of the algorithm comprehensively, an improved PSO algorithm with strong global search ability in the initial stage and strong local search ability in the later stage is needed. That is, the variation law of the inertia weight conforms to the decreasing law of being convex first and then concave, and the tansig function conforms well to this law.

Therefore, a tansig–NDIWAPSO for solving the modified electric field model is proposed, and the accurate identification of the Angle between the measurement axis of the 3D electronic compass and the measurement axis of the sensor was realized. By using the modified 3D electric field intensity directional decomposition model, the difficulty that insufficient measurement accuracy of electric field intensity component caused by the coaxial error between the electronic compass and the three measuring axes of the sensor is overcome, and a more accurate theoretical model is provided for the research of thunderstorm cloud orientation and lightning warning under the UAV-based mode.

## 2. Model and System of Atmospheric 3D Electric Field Orientation Measurement

A measurement system for the electric field carried by a UAV is proposed, which is composed of a grinding 3D electric field sensor, GPS module, 3D electronic compass, power module, and communication module. During the installation of the measuring system, three mutually orthogonal measuring axes X, Y, and Z in the sensor should be ensured to the maximum extent that they are parallel to the geographical east–west, geographical north–south, and plumb directions of the electronic compass, respectively. Under the action of the measured electric field, voltages $\left[U_{X},U_{Y},U_{Z}\right]$ are generated by three orthogonal induction electrodes, which $\left[U_{X},U_{Y},U_{Z}\right]$ are decoupled, and three orthogonal components $\left[{\vec{E}}_{X},{\vec{E}}_{Y},{\vec{E}}_{Z}\right]$ in the sensor’s local coordinate system (LSC) are obtained. However, in the process of measuring the aerial electric field, the sensor changes its position with the movement of the UAV, in order to obtain the geographical east–west, north–south and plumb direction electric field intensity components, it is necessary to transform the coordinates in LCS to the geographical coordinates, 3D electric field directional decomposition model in the geographic coordinates is shown in Equation (1).

(1) $$\left[\begin{array}{c} {\vec{E}}_{X}^{\prime }\\ {\vec{E}}_{Y}^{\prime }\\ {\vec{E}}_{Z}^{\prime } \end{array}\right]=\left[\begin{array}{ccc} \cos\theta & \sin\theta & 0\\ -\sin\theta & \cos\theta & 0\\ 0 & 0 & 1 \end{array}\right]\left[\begin{array}{ccc} \cos\beta & 0 & -\sin\beta \\ 0 & 1 & 0\\ \sin\beta & 0 & \cos\beta \end{array}\right]\left[\begin{array}{ccc} 1 & 0 & 0\\ 0 & \cos\alpha & \sin\alpha \\ 0 & -\sin\alpha & \cos\alpha \end{array}\right]\left[\begin{array}{c} {\vec{E}}_{X}\\ {\vec{E}}_{Y}\\ {\vec{E}}_{Z} \end{array}\right]$$

**Figure 1:**
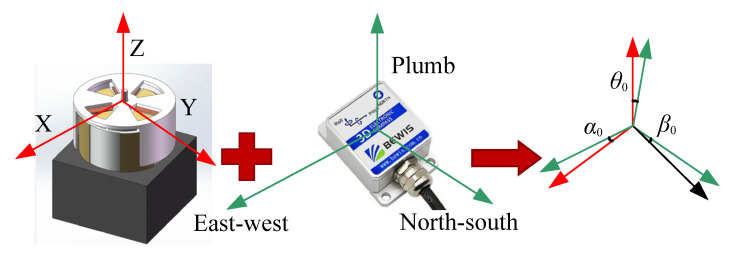
Angles between the three measuring axes of the electronic compass and three measuring axes of the sensor.

In Equation (1), α (pitch Angle), β (roll Angle), and θ (course Angle) are the angles between the three measuring axes of the electronic compass and the geographical east–west, geographical north–south, and plumb direction, respectively. However, in the process of system installation, the sensor and the electronic compass cannot achieve accurate coaxial parallel installation, and the two groups of orthogonal coordinate axes always have Angles in different directions. The Angles between the three measuring axes of the electronic compass and the three measuring axes of the sensor X, Y, and Z are $\alpha_{0}$, $\beta_{0}$, $\theta_{0}$, respectively, as shown in Figure 1.

Due to the included Angle $\alpha_{0}$, $\beta_{0}$, $\theta_{0}$, the 3D electric field directional decomposition model needs to be modified as shown in Equation (2).

(2) $$\left[\begin{array}{c} {\vec{E}}_{X}^{\prime \prime }\\ {\vec{E}}_{Y}^{\prime \prime }\\ {\vec{E}}_{Z}^{\prime \prime } \end{array}\right]=\left[\begin{array}{ccc} \cos\left(\theta +\theta_{0}\right) & \sin\left(\theta +\theta_{0}\right) & 0\\ -\sin\left(\theta +\theta_{0}\right) & \cos\left(\theta +\theta_{0}\right) & 0\\ 0 & 0 & 1 \end{array}\right]\left[\begin{array}{ccc} \cos(\beta +\beta_{0}) & 0 & -\sin(\beta +\beta_{0})\\ 0 & 1 & 0\\ \sin(\beta +\beta_{0}) & 0 & \cos(\beta +\beta_{0}) \end{array}\right]\left[\begin{array}{ccc} 1 & 0 & 0\\ 0 & \cos(\alpha +\alpha_{0}) & \sin(\alpha +\alpha_{0})\\ 0 & -\sin(\alpha +\alpha_{0}) & \cos(\alpha +\alpha_{0}) \end{array}\right]\left[\begin{array}{c} {\vec{E}}_{X}\\ {\vec{E}}_{Y}\\ {\vec{E}}_{Z} \end{array}\right]$$

**Figure 2:**
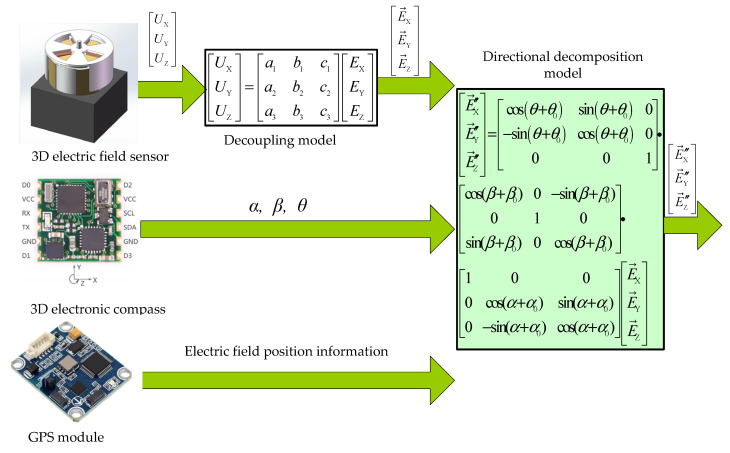
3D electric field directional measurement system.

In Equation (2), $\left[{\vec{E}}_{X}^{\prime \prime },{\vec{E}}_{Y}^{\prime \prime },{\vec{E}}_{Z}^{\prime \prime }\right]$ are the 3D electric field intensity components in the geographical coordinates. The modified model is a nonlinear equation set containing three parameters $\alpha_{0}$, $\beta_{0}$, $\theta_{0}$ to be identified. How to accurately solve $\alpha_{0}$, $\beta_{0}$, $\theta_{0}$ is the key to improving the accuracy of the modified model. The 3D electric field directional measurement system is shown in Figure 2.

## 3. Improved PSO Algorithm

### 3.1. Inertia Weight Impact on PSO

In view of the characteristics that the modified model shown in Equation (2), which is a nonlinear equation, an improved PSO algorithm is proposed to solve the model. The standard PSO algorithm updates the velocity and position of the particle according to Equation (3).

(3) $$\left\{\begin{array}{c} V_{k}^{t+1}=\omega V_{k}^{t}+c_{1}r_{1}(P_{k}^{t}-X_{k}^{t})+c_{2}r_{2}(P_{g}^{t}-X_{k}^{t})\\ X_{k}^{t+1}=X_{k}^{t}+V_{k}^{t+1} \end{array}\right.$$

In Equation (3), k is the *k*-th particle, t is the current number of iterations, $V_{k}^{t}$ and $V_{k}^{t+1}$ are the search velocities of the t and t+1 generations of the *k*-th particle, $X_{k}^{t}$ and $X_{k}^{t+1}$ are the positions of the t and t+1 generations of the *k*-th particle, $p_{k}^{t}$ is the optimal position of the *k*-th individual in generation t, $p_{g}^{t}$ is the optimal position of the global entity in generation t, $c_{1}$ are $c_{2}$ learning factors, $r_{1}$ and $r_{2}$ are two independent and uniformly distributed random numbers in [0, 1]. ω is the inertia weight of the velocity, The ideal search process of PSO algorithm is that the global search ability is strong in the initial stage, and the local search ability is strong in the later stage, A PSO algorithm with linear decreasing dynamic inertia weight (LDDIWPSO) is proposed [26], and the decreasing law is shown in the Equation (4)

(4) $$\omega_{t}=\omega_{\max}-\frac{(\omega_{\max}-\omega_{\min})\cdot t}{T_{\max}}$$

In Equation (4), $T_{\max}$ is the maximum iterations, $\omega_{\max}$ is the maximum inertia weight, and 0.95 is usually selected; $\omega_{\min}$ is the minimum inertia weight, and 0.4 is usually selected. The performance of the PSO algorithm is the best when ω decreases in concave function type, followed by linear function type, and the worst when convex function type. An exponentially decreasing dynamic inertia weight (EDDIWPSO) algorithm is proposed [27], and the change law of inertia weight adopts the exponential decreasing law, as shown in Equation (5).

(5) $$\omega_{t}=\omega_{\min}{\left(\frac{\omega_{\max}}{\omega_{\min}}\right)}^{\frac{1}{\left(1+u\frac{t}{T_{\max}}\right)}}$$

In Equation (5), if the value of u is too large, the algorithm will be premature and easily fall into the local optimal solution, and if u is too small, the value of $\omega_{t}$ will be much larger than $\omega_{\min}$ at the end of the iteration. Due to the value of u being difficult to determine, enlightened by the neural network Sigmoid function-tansig, so tansig–NDIWAPSO is proposed, as shown in Equation (6)

(6) $$\omega_{t}=\omega_{\max}-(\omega_{\max}-\omega_{\min})\cdot {\left(\frac{2}{1+e^{-\frac{a\cdot t}{t_{\max}}}}-1\right)}^{b}$$

**Figure 3:**
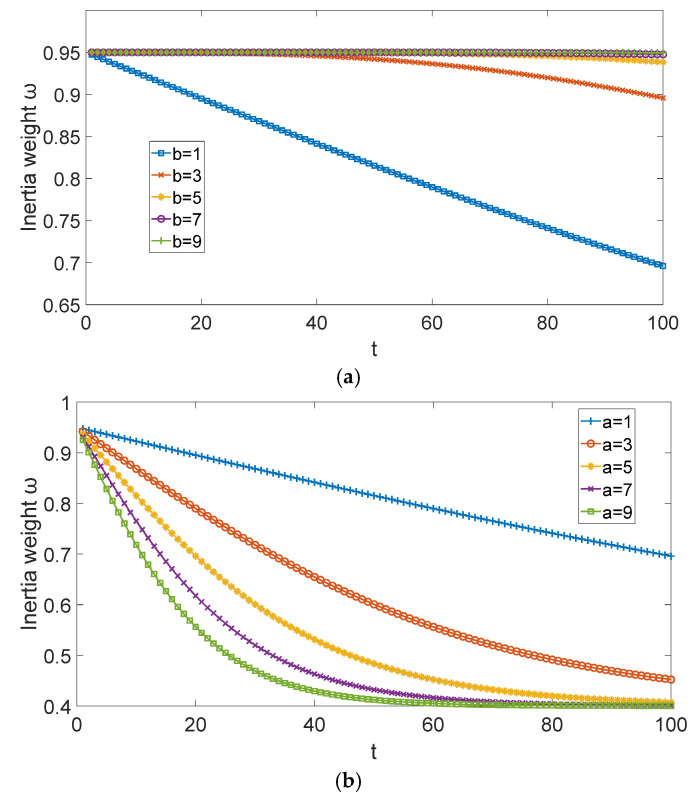
The influence of different a and b on ω: (**a**) the relationship between the convexity variation of ω and b; (**b**) the relationship between the concavity variation of ω and a.

In Equation (6), a and b are concavity and convexity adjustment factors for inertia weight, respectively. Set $\omega_{\max}=0.95$, $\omega_{\min}=0.4$, when a=1, the relationship between the convexity variation of ω and b is shown in the Figure 3a, when b=1, the relationship between the concavity variation of ω and a is shown in the Figure 3b.

**Figure 4:**
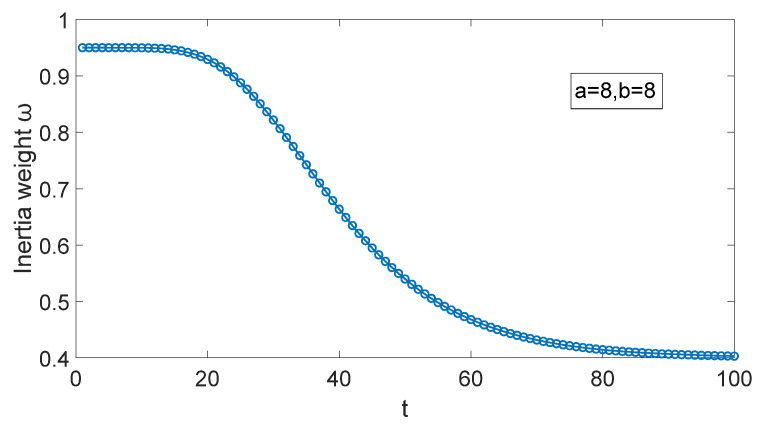
Change in the law of inertia weight $\omega_{t}$.

Since the initial value of $\omega_{t}$ is $\omega_{\max}$, it should be slowly reduced to maintain the global search ability, meanwhile avoid falling into the local optimal solution prematurely. In mid-iteration, $\omega_{t}$ needs to be decreased rapidly, the local search ability is improved, and the global search ability is reduced. In the late iteration, $\omega_{t}$ is decreased slowly to $\omega_{\min}$; the algorithm has a high local search ability, the convergence speed is improved. Therefore, in this paper, a=8, b=8, the change law of inertia weight $\omega_{t}$ is shown in Figure 4.

### 3.2. Example Simulation Analysis

Five algorithms, including the sparrow search algorithm (SSA) [28], improved cuckoo search algorithm (ICSA) [29], LDDIWPSO, EDDIWPSO and tansig–NDIWAPSO, were used respectively to test six commonly used test functions, the expressions, search space, and maximum speed of the six test functions are shown in Table 1. In the search space column of Table 1, upper right superscript represents the dimension of the search space. The dimension of the search space is 3 for F_1_ and F_2_, and the dimension of the search space is 10 for Sphere, Rosenbrock, Griewank and Rastrigin. Sphere and Rosenbrock are unimodal functions used to judge the convergence effect and convergence efficiency of the algorithm; Griewank and Rastrigin are multimodal functions used to test the case that the algorithm falls into a local optimal solution prematurely.

Algorithm parameters: In SSA, population size is 100, $T_{\max}=800$, the warning value is set to 0.6, the ratios of discoverers and participants are 0.7 and 0.3, respectively, and the ratio of those on alert is 0.2. In ICSA, $T_{\max}=800$, population size is 300. The remaining parameters were set in accordance with [29]. In LDDIWPSO, EDDIWPSO and tansig–NDIWAPSO. The initial values of the three improved PSO algorithms are set as $\omega_{\max}=0.95$, $\omega_{\min}=0.4$, population size is 300, $c_{1}=c_{2}=2$, $T_{\max}=800$, in EDDIWPSO, u=10, and in tansig–NDIWAPSO, a=8, b=8. On the basis of the above test parameters, 90 time-independent tests were run on the five different algorithms; the final test results are shown in Table 2.

According to Table 2, in in the optimization process of six different functions, compared with SSA, ICSA, LDDIWPSO and EDDIWPSO, tansig–NDIWAPSO can converge to the global optimal solution 0 within the range of iterations, and the global optimal solution is obtained with the least number of iterations, while the other four algorithms cannot converge to the global optimal solution within the range of the number of iterations. For the optimization process of Rosenbrock, the average fitness function value obtained by tansig–NDIWAPSO with the minimum average number of iterations (65) being 0.132. It is closer to the global optimal solution than the average fitness values of 3.383 (SSA), 1.556 (ICSA), 2.523 (LDDIWPSO), and 1.324 (EDDIWPSO) obtained by the other four algorithms. Moreover, compared with the variance of the fitness values of the other four algorithms, it has the least errors and the best stability.

### 3.3. Parameter Identification Method of 3D Electric Field Directional Decomposition Modified Model Based on Tansig–NDIWAPSO Algorithm

The PSO is used to solve the modified model of 3D electric field directional decomposition, which can be regarded as the problem of minimizing the error between ${\vec{E}}_{X}^{\prime \prime }$, ${\vec{E}}_{Y}^{\prime \prime }$, ${\vec{E}}_{Z}^{\prime \prime }$, and actual loaded electric field intensity components ${\vec{E}}_{X}^{\left(0\right)}$, ${\vec{E}}_{Y}^{\left(0\right)}$, ${\vec{E}}_{Z}^{\left(0\right)}$. The fitness objective function F of the PSO algorithm is shown in Equation (7).

(7) $$F=\left|f_{1}\right|+\left|f_{2}\right|+\left|f_{3}\right|=\left|{\vec{E}}_{X}^{\prime \prime }-{\vec{E}}_{X}^{\left(0\right)}\right|+\left|{\vec{E}}_{Y}^{\prime \prime }-{\vec{E}}_{Y}^{\left(0\right)}\right|+\left|{\vec{E}}_{Z}^{\prime \prime }-{\vec{E}}_{Z}^{\left(0\right)}\right|$$

In Equation (7),

$$\left[\begin{array}{c} f_{1}\\ f_{2}\\ f_{3} \end{array}\right]=\left[\begin{array}{ccc} \cos\left(\theta +\theta_{0}\right) & \sin\left(\theta +\theta_{0}\right) & 0\\ -\sin\left(\theta +\theta_{0}\right) & \cos\left(\theta +\theta_{0}\right) & 0\\ 0 & 0 & 1 \end{array}\right]\left[\begin{array}{ccc} \cos(\beta +\beta_{0}) & 0 & -\sin(\beta +\beta_{0})\\ 0 & 1 & 0\\ \sin(\beta +\beta_{0}) & 0 & \cos(\beta +\beta_{0}) \end{array}\right]\left[\begin{array}{ccc} 1 & 0 & 0\\ 0 & \cos(\alpha +\alpha_{0}) & \sin(\alpha +\alpha_{0})\\ 0 & -\sin(\alpha +\alpha_{0}) & \cos(\alpha +\alpha_{0}) \end{array}\right]\left[\begin{array}{c} {\vec{E}}_{X}\\ {\vec{E}}_{Y}\\ {\vec{E}}_{Z} \end{array}\right]-\left[\begin{array}{c} {\vec{E}}_{X}^{\left(0\right)}\\ {\vec{E}}_{Y}^{\left(0\right)}\\ {\vec{E}}_{Z}^{\left(0\right)} \end{array}\right]$$

${\vec{E}}_{X}^{\left(0\right)}$, ${\vec{E}}_{Y}^{\left(0\right)}$, ${\vec{E}}_{Z}^{\left(0\right)}$ are generated by adjusting the voltage of the parallel plate capacitor and the position of the measuring system in the electric field; ${\vec{E}}_{X}$, ${\vec{E}}_{Y}$, ${\vec{E}}_{Z}$ are obtained by the decoupling matrix shown in Equation (8).

(8) $$\left[\begin{array}{c} {\vec{E}}_{X}\\ {\vec{E}}_{Y}\\ {\vec{\vec{E}}}_{Z} \end{array}\right]=C\left[\begin{array}{c} U_{X}\\ U_{Y}\\ U_{Z} \end{array}\right]=\left[\begin{array}{ccc} 36.3665 & -10.019 & -1.07461\\ -7.85491 & 37.9304 & -31.5856\\ -6.84204 & -11.9843 & 103.8711 \end{array}\right]\cdot \left[\begin{array}{c} U_{X}\\ U_{Y}\\ U_{Z} \end{array}\right]$$

**Figure 5:**
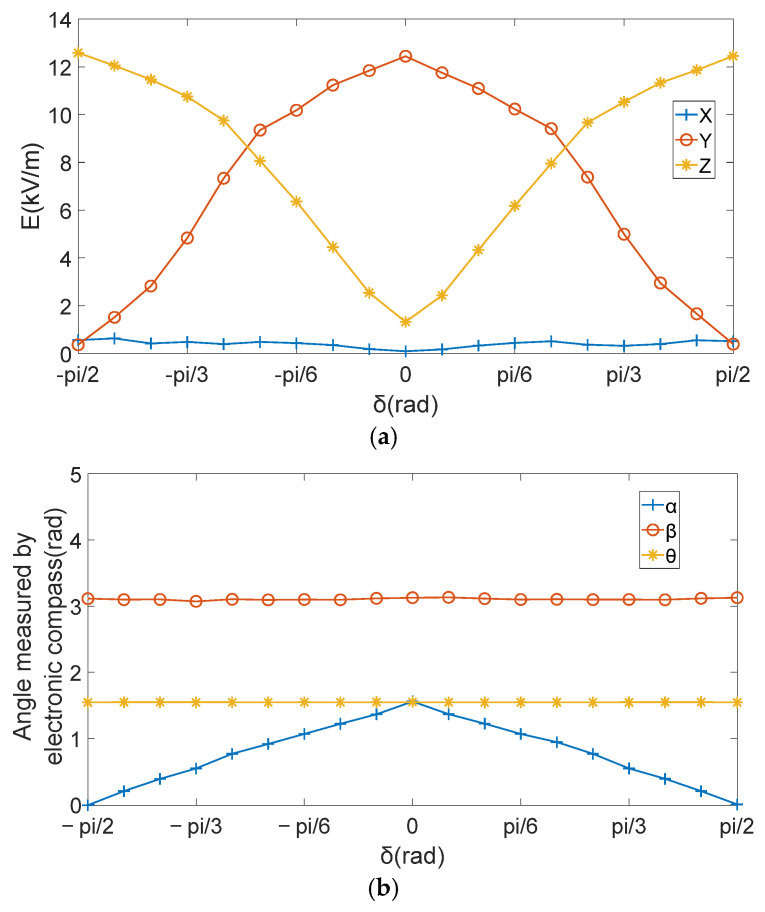
Experimental data record. (**a**) Electric field intensity $\left[{\vec{E}}_{X},{\vec{E}}_{Y},{\vec{E}}_{Z}\right]$. (**b**) Rotated angle $\left[\alpha ,\beta ,\theta \right]$.

**Figure 6:**
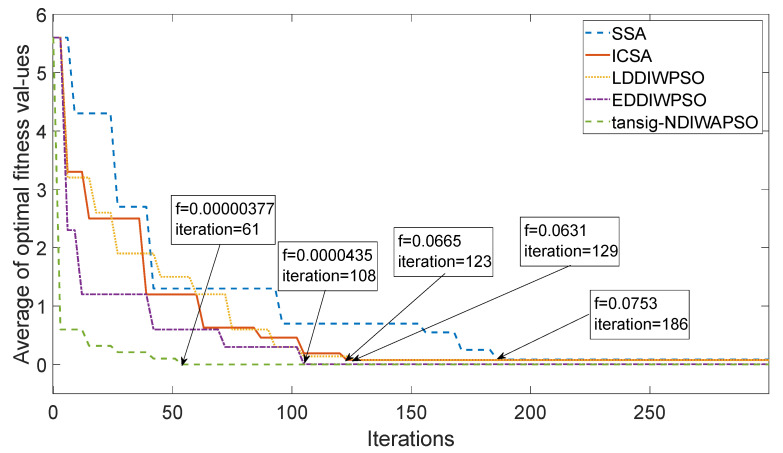
Iterative curve for optimizing fitness.

In Equation (8), C is the decoupling matrix, $\left[U_{X},U_{Y},U_{Z}\right]$, which were obtained by the sensor. The measurement system of a 3D electric field adopts a modular design approach, which is mainly comprised of a GPS, 3D electric compass, power supply, and wireless transmission device. In addition to the self-developed three-dimensional field friction sensor, the structure of the three-dimensional electric field measurement system, as shown in reference [14], also includes modules such as GPS, 3D electronic compass, power supply, and wireless transmission [18]. Among them, the GPS adopts BN-220, and the power supply adopts a multi-channel power supply system to meet the requirements of different modules. The 3D electronic compass adopts the attitude reference system (AHRS) nine-axis inertial navigation sensor, the wireless transmission uses E61-TTL-1W, and the data processing module adopts the STM32F103RC single-chip microcomputer. The optimized process of $\alpha_{0}$, $\beta_{0}$, $\theta_{0}$ is as follows:

(1) The measurement system is fixed on the rotating experimental platform, and the experimental platform is placed in a pre-adjusted uniform electric field;
(2) By adjusting the position of the rotating platform, the initial position of the electronic compass is α=β=θ=0;
(3) By adjusting the power supply, the uniform electric field intensity is a constant value $\left[{\vec{E}}_{X}^{\left(0\right)}=0,{\vec{E}}_{Y}^{\left(0\right)}=0,{\vec{E}}_{Z}^{\left(0\right)}=12\mathrm{kV}/m\right]$;
(4) Make the platform rotate around the geographical east–west axis (rotated in the plane perpendicular to the geographical east–west); the rotation Angle δ is the Angle between the rotating platform and the horizontal plane, δ=[−π/2,π/2]. The voltage obtained by the sensor’s three induction electrodes $\left[U_{X},U_{Y},U_{Z}\right]$ and the Angles $\left[\alpha ,\beta ,\theta \right]$ were recorded, $\left[{\vec{E}}_{X},{\vec{E}}_{Y},{\vec{E}}_{Z}\right]$ were obtained by matrix decoupling. The result is shown in Figure 5.

(5) The experimental measurement data were taken as a group $\left[{\vec{E}}_{X},{\vec{E}}_{Y},{\vec{E}}_{Z},\alpha ,\beta ,\theta \right]$, and each of the three groups of measurement data were substituted into the 3D electric field directional decomposition modified model, the equations were solved with SSA, ICSA, LDDIWPSO, EDDIWPSO and tansig–NDIWAPSO, respectively. In SSA, the parameter Settings should be made in accordance with [28]; in ICSA, the parameter Settings should be made in accordance with [29], in LDDIWPSO, EDDIWPSO and tansig–NDIWAPSO, the initial inertia weight of the three improved PSO algorithms are $\omega_{\max}=0.95$, $\omega_{\min}=0.4$, population size is 100, $c_{1}=c_{2}=2$, $T_{\max}=300$, in EDDIWPSO, u=10, in tansig–NDIWAPSO, a=8, b=8. The iterative optimization curves of the fitness function values of the SSA, ICSA, LDDIWPSO, EDDIWPSO and tansig–NDIWAPSO are shown in Figure 6. Five algorithms were independently run 50 times, and the results of the six methods are shown in Table 3.

As shown in Figure 6 and Table 3, compared with the other four algorithms, when tansig–NDIWAPSO is used to solve the modified model of 3D electric field decomposition, the average optimal fitness value $3.77\times 10^{-5}$ can be obtained within the average minimum iterations (61 generations). The average optimal fitness value obtained by the other four algorithms are $7.53\times 10^{-2}$, $6.04\times 10^{-2}$, $6.31\times 10^{-2}$, and $4.35\times 10^{-5}$, and more iterations are needed (186, 123, 129, and 108).

## 4. Accuracy Verification Experiment and Result Analysis of the Modified Model

**Figure 7:**
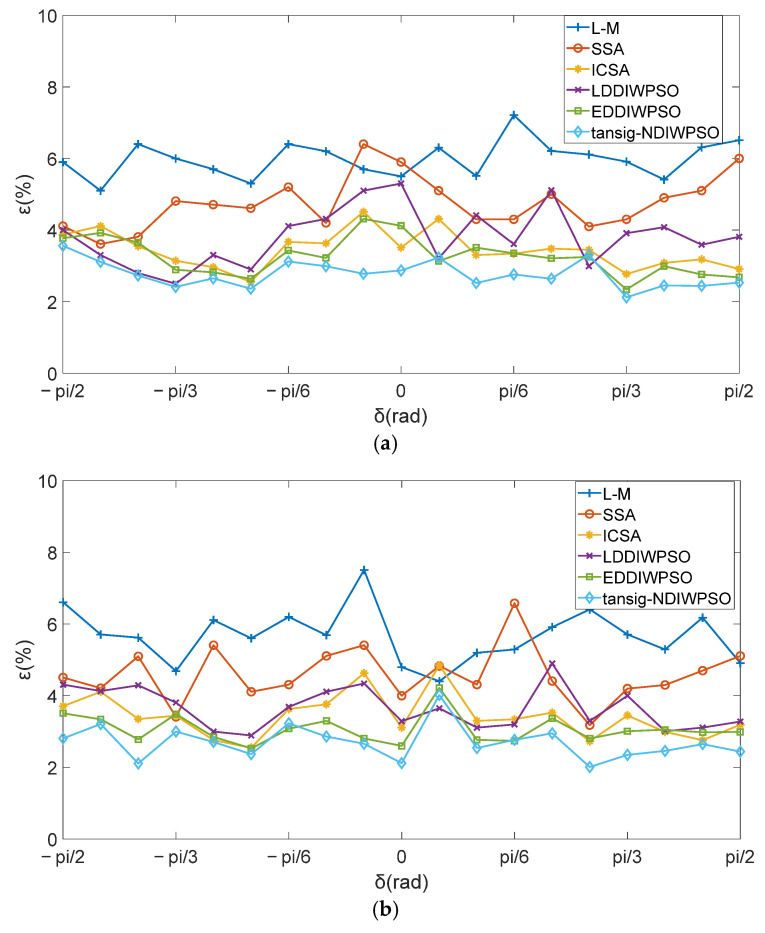

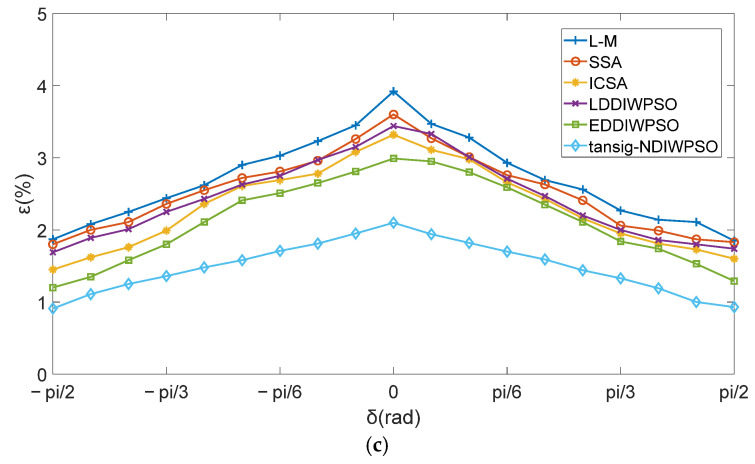
The relative error ε of the electric field component in different directions: (**a**) geographical east–west; (**b**) geographical north–south; and (**c**) geographical plumb.

**Figure 8:**
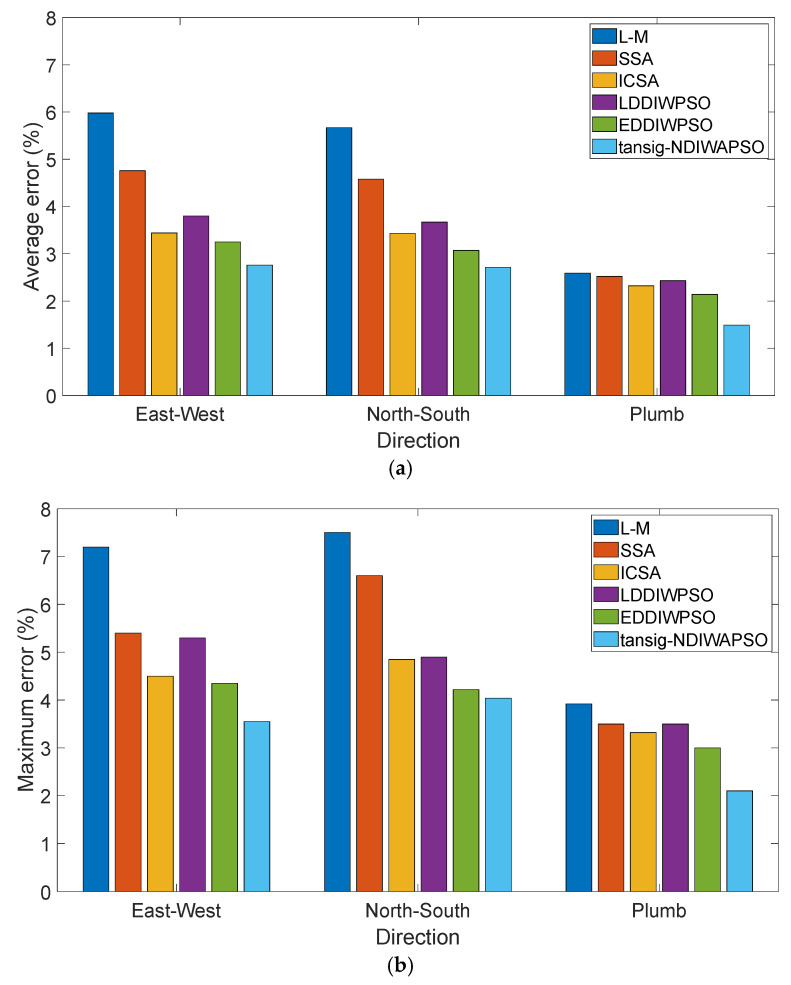
Average and maximum errors in different directions: (**a**) average errors; (**b**) maximum errors.

The $\left[\alpha_{0},\beta_{0},\theta_{0}\right]$ obtained by the Levenberg–Marquardt (L-M) [18], SSA, ICSA, LDDIWPSO, EDDIWPSO and tansig–NDIWAPSO algorithms were substituted into the modified model of 3D electric field directional decomposition; respectively, six different directional decomposition modified models were obtained, with each named after the corresponding algorithm as L-M, SSA, ICSA, LDDIWPSO, EDDIWPSO, and tansig–NDIWAPSO. The experimental data $\left[{\vec{E}}_{X},{\vec{E}}_{Y},{\vec{E}}_{Z},\alpha ,\beta ,\theta \right]$, as shown in Figure 7, were substituted into the six different models. The intensity components of the 3D electric field along the geographical east–west, north–south, and plumb directions $\left[{\vec{E}}_{X}^{\prime \prime },{\vec{E}}_{Y}^{\prime \prime },{\vec{E}}_{Z}^{\prime \prime }\right]$ were obtained. ${\vec{E}}_{X}^{\prime \prime }$, ${\vec{E}}_{Y}^{\prime \prime }$, ${\vec{E}}_{Z}^{\prime \prime }$ are compared with the actual loaded electric field intensity ${\vec{E}}_{X}^{\left(0\right)}=0$, ${\vec{E}}_{Y}^{\left(0\right)}=0$, ${\vec{E}}_{Z}^{\prime \prime (0)}=12$, respectively. The relative error ε between the ${\vec{E}}_{X}^{\prime \prime }$, ${\vec{E}}_{Y}^{\prime \prime }$, ${\vec{E}}_{Z}^{\prime \prime }$ and ${\vec{E}}_{X}^{\left(0\right)}$, ${\vec{E}}_{Y}^{\left(0\right)}$, ${\vec{E}}_{Z}^{\left(0\right)}$, and the change in error ε as the δ change is recorded, as shown in Figure 7, the average and maximum errors are shown in Figure 8. Since ${\vec{E}}_{X}^{\left(0\right)}={\vec{E}}_{Y}^{\left(0\right)}=0$, these two directions (geographical east–west and geographical north–south) are represented by quote errors ε in different directions as shown in Equation (9).

(9) $$\varepsilon =\left\{\begin{array}{c} \left|\frac{{\vec{E}}_{d}^{\prime \prime }}{\max({\vec{E}}_{d}^{\left(0\right)})}\right|,d=\mathrm{East}\text{-}\mathrm{west},\;\mathrm{North}\text{-}\mathrm{South}\\ \left|\frac{\left|{\vec{E}}_{Z}^{\prime \prime }-\max({\vec{E}}_{d}^{\left(0\right)})\right|}{\max({\vec{E}}_{d}^{\left(0\right)})}\right|,d=\mathrm{Plump}, \end{array}\right.,\max({\vec{E}}_{d}^{\left(0\right)})=12$$

The results shown in Figure 7 and Figure 8 were analyzed in terms of the following four aspects:

(1) The effect of models obtained by different algorithms on measurement errors.

As can be seen from Figure 7, compared with other algorithms, the model is obtained by using the algorithm proposed in this paper, the the fewest errors are between ${\vec{E}}_{X}^{\prime \prime }$, ${\vec{E}}_{Y}^{\prime \prime }$, ${\vec{E}}_{Z}^{\prime \prime }$. As shown in Figure 8a, in the geographical east–west direction, the average errors between ${\vec{E}}_{X}^{\prime \prime }$ and ${\vec{E}}_{X}^{\left(0\right)}$ of the six models are 5.98%, 4.76%, 3.44%, 3.80%, 3.26%, and 2.77%, respectively; in the geographical north–south direction, the average errors between ${\vec{E}}_{Y}^{\prime \prime }$ and ${\vec{E}}_{Y}^{\left(0\right)}$ of the six models are 5.67%, 4.58%, 3.43%, 3.67%, 3.07% and 2.70% respectively; in the geographical plumb direction, the average errors between ${\vec{E}}_{Z}^{\prime \prime }$ and ${\vec{E}}_{Z}^{\left(0\right)}$ of the six models are 2.68%, 2.52%, 2.32%, 2.43%, 2.14% and 1.49%, respectively. The maximum errors of the six models in three different directions are compared, as shown in Figure 8b. In the geographical east–west direction, the maximum errors are 7.2%, 6.4%, 4.51%, 5.31%, 4.33%, and 3.56%, respectively; in the geographical north–south direction, the errors are 7.5%, 6.6%, 4.85%, 4.9%, 4.22%, and 4.04%, respectively; in the geographical plumb direction, the errors are 3.92%, 3.6%, 3.32%, 3.49%, 3.01%, and 2.10% respectively. Therefore, in either direction, when the tansig–NDIWAPSO is used to solve the modified model of 3D electric field directional decomposition, the obtained $\left[\alpha_{0},\beta_{0},\theta_{0}\right]$ is closer to the true value, the relative error ε be ${\vec{E}}_{X}^{\prime \prime }$, ${\vec{E}}_{Y}^{\prime \prime }$, ${\vec{E}}_{Z}^{\prime \prime }$ are closer to the ${\vec{E}}_{X}^{\left(0\right)}$, ${\vec{E}}_{Y}^{\left(0\right)}$, ${\vec{E}}_{Z}^{\left(0\right)}$, and the modified 3D electric field directional decompositions model which was solved by tansig–NDIWAPSO is more accurate.

(2) For the same model, the measurement errors in different directions are quite different.

As shown in Figure 7, no matter which algorithm is used to obtain the model, the errors in the geographical east–west direction and in the geographical north–south direction are relatively large, and the two are relatively close, and the error in the geographical plumb direction is the least. Taking the model obtained by the tansig–NDIWAPSO algorithm as an example, the average errors in the geographical east–west, geographical north–south and plumb directions are 2.77%, 2.70%, and 1.49%, respectively, and the maximum errors are 3.56%, 4.04%, and 2.1%, respectively. The main reason for this phenomenon is: ${\vec{E}}_{X}^{\left(0\right)}=0$, ${\vec{E}}_{Y}^{\left(0\right)}=0$, ${\vec{E}}_{Z}^{\left(0\right)}=12$, no electric fields are loaded in the geographical east–west and north–south direction, the electric field intensity ${\vec{E}}_{X}^{\prime \prime }$ and ${\vec{E}}_{Y}^{\prime \prime }$ obtained by the model are mainly generated by the coupling effect, which is caused by electric field distortion. Due to the coupling effect, and ${\vec{E}}_{Z}^{\prime \prime }$ and ${\vec{E}}_{Y}^{\prime \prime }$ are uncertain. However, there is an electric field ${\vec{E}}_{Z}^{\left(0\right)}=12$ loading in the geographical plumb direction—the measurement error is the least in this direction.

(3) For the same model, the measurement errors ε in different directions have different laws as δ changes.

According to Figure 7a,b, in the geographical east–west, north–south direction, there is no obvious law in error distribution. Since the electric field is actually loaded in the plumb direction, the electric field intensity in these two directions is caused by ${\vec{E}}_{Z}^{\left(0\right)}$; ${\vec{E}}_{Z}^{\left(0\right)}=12$ is a constant value, the coupling component is also relatively stable, so the errors have no obvious change law.

Compared with Figure 7a,b, no matter which algorithm is used to modify the model in Figure 7c, in the geographical plumb direction, the error between ${\vec{E}}_{Z}^{\prime \prime }$ and ${\vec{E}}_{Z}^{\left(0\right)}$ shows a certain rule with the change of δ. When δ=π/2 or δ=−π/2, the error is minimum, and when δ=0, the error is maximum. When δ is within the range of $\left[-\pi /2,\pi /2\right]$. The closer δ is to 0, the greater the error, and conversely, the farther away from 0, the less the error.

The reason for this phenomenon is that the geographical plumb direction is the actual electric field loading direction, according to the modified electric field directional decomposition model, when $\left[\alpha_{0},\beta_{0},\theta_{0}\right]$ is determined, $\left[{\vec{E}}_{X}^{\prime \prime },{\vec{E}}_{Y}^{\prime \prime },{\vec{E}}_{Z}^{\prime \prime }\right]$ is determined by $\left[{\vec{E}}_{X},{\vec{E}}_{Y},{\vec{E}}_{Z},\alpha ,\beta ,\theta \right]$, especially ${\vec{E}}_{Z}^{\prime \prime }$, for which the mathematical model is shown in Equation (10):

(10) $${\vec{E}}_{Z}^{\prime \prime }={\vec{E}}_{X}\sin(\beta +\beta_{0})-{\vec{E}}_{Y}\cos(\beta +\beta_{0})\sin(\alpha +\alpha_{0})+{\vec{E}}_{Z}\cos(\beta +\beta_{0})\cos(\alpha +\alpha_{0})$$

According to the Angle changes shown in Figure 5b, the roll Angle β and course Angle θ tend to be relatively stable; β is close π/2, θ is close to π, and $\alpha_{0},\beta_{0},\theta_{0}$ is relatively small, which are close to 0. In order to analyze its change law, the modified model can be simplified, as shown in Equation (11):

(11) $${\vec{E}}_{Z}^{\prime \prime }={\vec{E}}_{Y}\sin\alpha -{\vec{E}}_{Z}\cos\alpha$$

In Equation (11), when α is near π/2 or −π/2, ${\vec{E}}_{Z}^{\prime \prime }$ is greatly influenced by ${\vec{E}}_{Y}$, and when α is near 0, ${\vec{E}}_{Z}^{\prime \prime }$ is greatly influenced by ${\vec{E}}_{Z}$, he variation trend of pitch Angle α is roughly almost with that of platform rotation Angle δ. Therefore, when α is near 0, ${\vec{E}}_{Z}^{\prime \prime }$ is mainly measured by the fan-shaped planar induction electrode on the top of the sensor; while α is far from 0, ${\vec{E}}_{Z}^{\prime \prime }$ is mainly measured by the cylindrical induction electrode on the side of the sensor.

**Figure 9:**
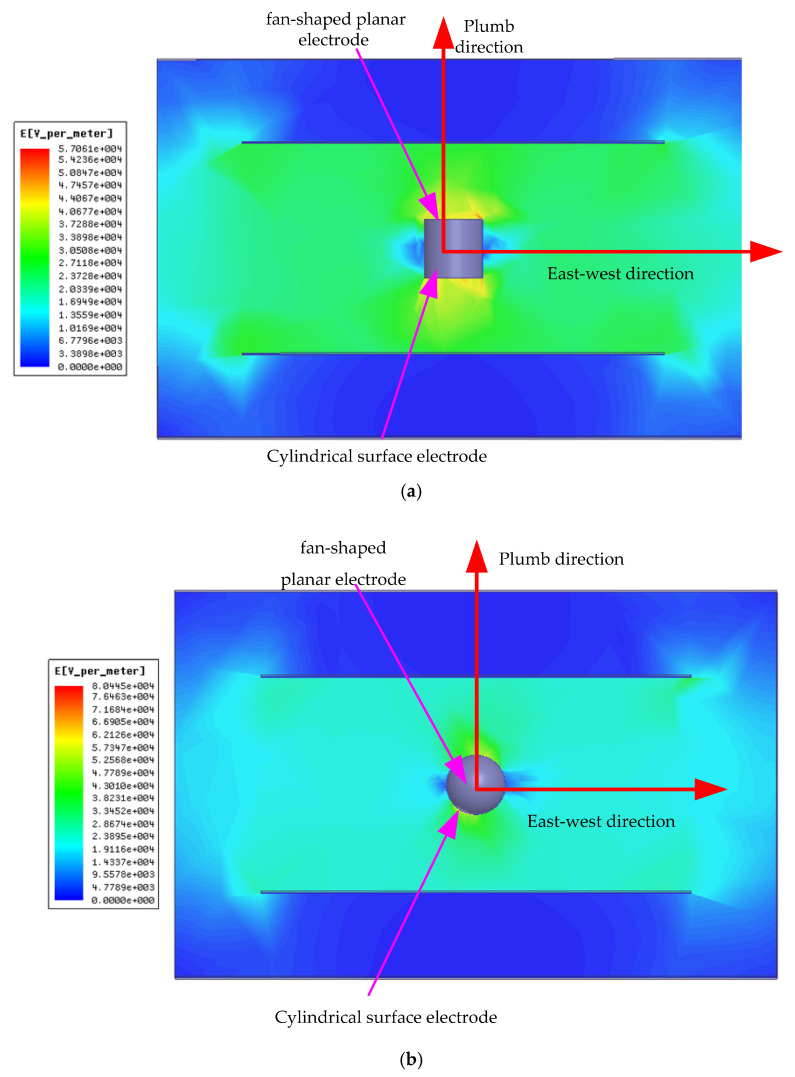
Electric field distortion effect caused by sensor: (**a**) δ=0; (**b**) δ=π/2 or δ=−π/2.

Due to the different shapes of the induction electrodes in each direction of the sensor, the degrees of electric field distortion in the three orthogonal directions are also different. Ansoft Maxwell 16.0 was used to simulate the electric field coupling effect. As shown in Figure 9a, when δ=0, the fan-shaped planar electrode of the sensor is perpendicular to the geographic plumb direction (the direction of electric field loading). The planar electrode brings about a significant distortion effect on the electric field, and the cylindrical electrode has little distortion on the electric field. In Figure 9b, when δ=π/2 or δ=−π/2, the fan-shaped plane of the sensor is perpendicular to the geographic north–south direction, the intensity is mainly measured by the sensor’s cylindrical electrode. The cylindrical electrode causes less distortion, which leads to the measurement accuracy being higher than that when δ=0, resulting in the change rule as shown in Figure 7c.

(4) The influence of the algorithm on the model accuracy.

By analyzing the mean square error (MSE) and variance of the intensity components obtained by six different models, the accuracy of the models obtained by six different algorithms was verified. The variance and MSE (mean-square error) are shown in Equation (12):

(12) $$\left\{\begin{array}{c} \mathrm{MSE}=\frac{1}{n}\sum_{i=1}^{n}{\left(\left|{\vec{E}}_{d}^{\prime \prime }\right|-\left|{\vec{E}}_{d}^{\left(0\right)}\right|\right)}^{2}\\ \sigma =\frac{1}{n}\sum_{i=1}^{n}{\left(\left|{\vec{E}}_{d}^{\prime \prime }\right|-\mathrm{AVERAGE}(\left|{\vec{E}}_{d}^{\prime \prime }\right|)\right)}^{2} \end{array}\right.$$

**Figure 10:**
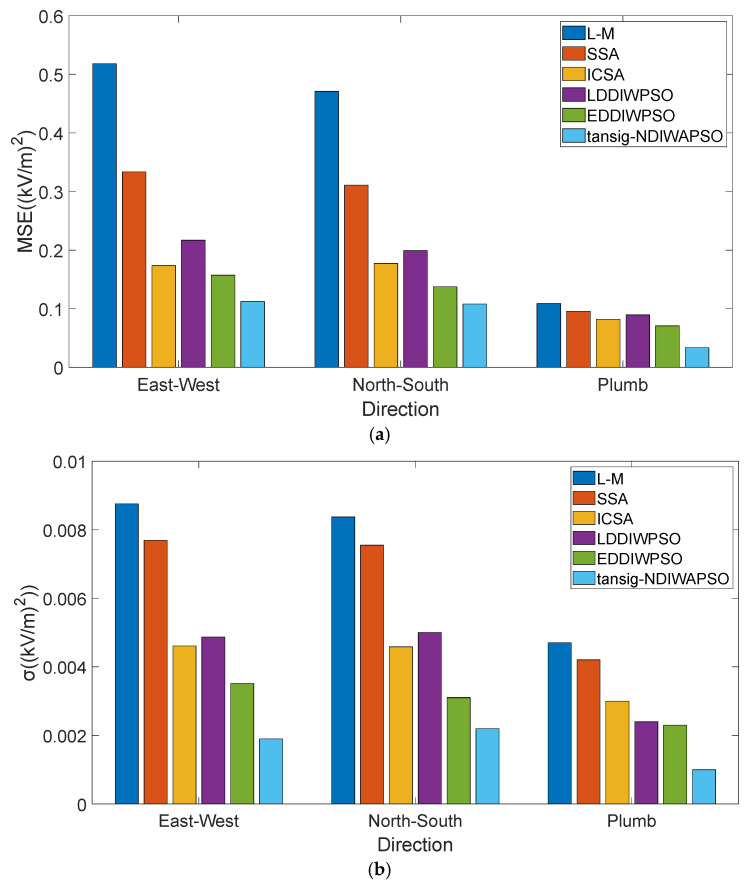
MSE and σ of $\left[{\vec{E}}_{X}^{\prime \prime },{\vec{E}}_{Y}^{\prime \prime },{\vec{E}}_{Z}^{\prime \prime }\right]$: (**a**) MSE; (**b**) σ.

In Equation (12), σ is the variance, AVERAGE () is the average function for the average of $\left|{\vec{E}}_{d}^{\prime \prime }\right|$ calculated. n is the sample size of the model. The MSE and σ of $\left[{\vec{E}}_{X}^{\prime \prime },{\vec{E}}_{Y}^{\prime \prime },{\vec{E}}_{Z}^{\prime \prime }\right]$ obtained by the six models are shown in Figure 10, in the geographical east–west or north–south direction, ${\vec{E}}_{d}^{\left(0\right)}=0$, and in the plumb direction ${\vec{E}}_{d}^{\left(0\right)}=12$.

In Figure 10a, the MSE of $\left[{\vec{E}}_{X}^{\prime \prime },{\vec{E}}_{Y}^{\prime \prime },{\vec{E}}_{Z}^{\prime \prime }\right]$, by comparing the above data, the MSE obtained by the model obtained by the tansig–NDIWAPSO algorithm, is the minimum (0.1122, 0.1081, 0.0335) in three directions. The obtained value is closer to the real value ${\vec{E}}_{X}^{\left(0\right)}=0$, ${\vec{E}}_{Y}^{\left(0\right)}=0$, ${\vec{E}}_{Z}^{\left(0\right)}=12$, and the accuracy of the model obtained by the tansig–NDIWAPSO algorithm is higher.

To further verify the accuracy of the model obtained by the tansig–NDIWAPSO algorithm, the variances of $\left[{\vec{E}}_{X}^{\prime \prime },{\vec{E}}_{Y}^{\prime \prime },{\vec{E}}_{Z}^{\prime \prime }\right]$ obtained by the six models are analyzed. As shown in Figure 10b, the σ of $\left[{\vec{E}}_{X}^{\prime \prime },{\vec{E}}_{Y}^{\prime \prime },{\vec{E}}_{Z}^{\prime \prime }\right]$ obtained by the tansig–NDIWAPSO algorithm in three directions are 0.0019, 0.0022, and 0.0011, respectively, compared with the other five models, σ which is the least in each of the three directions for the model obtained by the tansig–NDIWAPSO algorithm. It is proven that the model obtained by the tansig–NDIWAPSO algorithm has higher precision, the accuracy of tansig–NDIWAPSO in optimizing the modified model is proved, and tansig–NDIWAPSO has better stability and convergence.

## 5. Conclusions

Based on the establishment of a modified 3D electric field directional decomposition model including the Angles between three measuring axes of the sensor and electronic compass, considering the characteristics of the model as a nonlinear equation system, a tansig–NDIWAPSO algorithm is proposed to identify the model parameters (including Angles), a more accurate group of angles and a modified model are obtained. The modified model optimized by six different algorithms, such as tansig–NDIWAPSO and so on, is used for directional measurement in the electrostatic field environment simulation and rotation calibration experiment. The simulation and experimental results show that:

(1) By adopting the tansig–NDIWAPSO to solve the modified 3D electric field directional decomposition, the obtained $\left[\alpha_{0},\beta_{0},\theta_{0}\right]$ is closer to the true angle. The $\left[{\vec{E}}_{X}^{\prime \prime },{\vec{E}}_{Y}^{\prime \prime },{\vec{E}}_{Z}^{\prime \prime }\right]$ obtained by the modified model are closer to the actual loading $\left[{\vec{E}}_{X}^{\left(0\right)},{\vec{E}}_{Y}^{\left(0\right)},{\vec{E}}_{Z}^{\left(0\right)}\right]$, and the measured results are more accurate.
(2) The coupling effect exerts varying influences on measurement error in different directions. In the direction orthogonal to the electric field, the coupling effect has a more significant impact on the measurement error, while in the direction of the electric field, the coupling effect has a relatively minor influence.
(3) When the modified model of directional decomposition was determined, by analyzing the theoretical model of intensity in loading direction, two methods, the electrostatic field simulation based on Ansoft Maxwell and the rotated calibrating experiment, were adopted respectively, and the electric field distortion caused by the planar induction electrode is greater than that of the cylindrical surface is effectively verified.
(4) By analyzing the MSE and variance of $\left[{\vec{E}}_{X}^{\prime \prime },{\vec{E}}_{Y}^{\prime \prime },{\vec{E}}_{Z}^{\prime \prime }\right]$ obtained from the six models, it is verified that the model obtained by using the tansig–NDIWAPSO has higher accuracy, and the stability and convergence of the tansig–NDIWAPSO are superior, which can further enhance the measurement accuracy of the 3D electric field decomposition in the geographic coordinates, a more accurate theoretical model for realizing the orientation of thunderstorm cloud in the mode of sensor mounted by UAV.

## Figures and Tables

**Table 1:** Test functions.

Function	Function Expression	Search Space	$V_{\max}$	Global Minimum
F_1_	$\begin{array}{l} x_{1}^{3}-x_{1}x_{2}x_{3}=0\\ x_{2}^{2}-x_{1}x_{3}=0\\ 10x_{1}x_{2}x_{3}-x_{1}-0.1=0 \end{array}$	[−5, 5]^3^	10	/
F_2_	$\begin{array}{l} e^{x_{1}^{2}}-8x_{1}=0\\ x_{1}+x_{2}-1=0\\ {\left(x_{3}-1\right)}^{3}=0 \end{array}$	[−5, 5]^3^	10	/
Sphere	$f_{1}(x)=\sum_{i=1}^{D}x_{i}^{2}$	[−100, 100]^10^	100	0
Rosenbrock	$f_{2}(x)=\sum_{i=1}^{D}\left[100{\left(x_{i+1}-x_{i}\right)}^{2}+{\left(x_{i}-1\right)}^{2}\right]$	[−100, 100]^10^	100	0
Griewank	$f_{3}(x)=\frac{1}{4000}\sum_{i=1}^{D}(x_{i}^{2})-\prod_{i=1}^{D}\cos(\frac{x_{i}}{\sqrt{i}})+1$	[−600, 600]^10^	600	0
Rastrigrin	$f_{4}(x)=\sum_{i=1}^{D}\left[x_{i}^{2}-10\cos(2\pi x_{i})+10\right]$	[−10, 10]^10^	10	0

**Table 2:** Final test results.

Function	Optimization Method	Average of Optimal Values	Variance of the Optimal Value	Average Number of Iterations
F_1_	SSA	$3.006\times 10^{-5}$	$6\times 10^{-7}$	>300
	ICSA	0	0	74
	LDDIWPSO	0	0	102
	EDDIWPSO	0	0	58
	tansig–NDIWAPSO	0	0	45
F_2_	SSA	$2.993\times 10^{-5}$	$4\times 10^{-7}$	>300
	ICSA	$1.997\times 10^{-7}$	0	55
	LDDIWPSO	0	0	172
	EDDIWPSO	0	0	65
	tansig–NDIWAPSO	0	0	38
Sphere	SSA	$7.873\times 10^{-5}$	$1.187\times 10^{-6}$	>800
	ICSA	$4.134\times 10^{-6}$	$1.037\times 10^{-6}$	>500
	LDDIWPSO	$7.435\times 10^{-6}$	$8.659\times 10^{-6}$	>500
	EDDIWPSO	$3.254\times 10^{-10}$	$1.32\times 10^{-10}$	>300
	tansig–NDIWAPSO	0	0	73
Rosenbrock	SSA	3.383	0.832	>500
	ICSA	1.556	0.531	>300
	LDDIWPSO	2.531	0.649	>300
	EDDIWPSO	1.324	0.436	>300
	tansig–NDIWAPSO	0.132	0.024	58
Griewank	SSA	0.0613	0.00319	>500
	ICSA	0.0383	0.00218	>300
	LDDIWPSO	0.0465	0.00247	>800
	EDDIWPSO	0	0	70
	tansig–NDIWAPSO	0	0	55
Rastrigrin	SSA	1.574	0.282	>600
	ICSA	1.398	0.185	>500
	LDDIWPSO	1.536	0.216	>600
	EDDIWPSO	1.127	0.112	236
	tansig–NDIWAPSO	0	0	62

**Table 3:** Results by different methods.

Algorithms	Solution of Equations			Average of Optimal Fitness Values	Average Number of Iterations
	$\alpha_{0}$	$\beta_{0}$	$\theta_{0}$		
SSA	−0.01871	0.02454	0.01365	$7.53\times 10^{-2}$	186
ICSA	−0.02134	0.02773	0.01231	$6.04\times 10^{-2}$	123
LDDIWPSO	−0.01856	0.02851	0.01402	$6.31\times 10^{-2}$	129
EDDIWPSO	−0.01647	0.03145	0.01375	$4.35\times 10^{-5}$	108
tansig–NDIWAPSO	−0.01368	0.03318	0.01176	$3.77\times 10^{-5}$	61

## Data Availability

The data are available from the corresponding author on reasonable request.

## Abbreviations

The following abbreviations are used in this manuscript:

3D	Three-dimensional
UAV	Unmanned aerial vehicle
PSO	Particle swarm optimization
L-M	Levenberg–Marquardt
ICSA	Improved cuckoo search algorithm
SSA	Sparrow search algorithm
NDIWAPSO	Nonlinear dynamic inertia weight adaptive particle swarm optimization
MEMS	Micro-electromechanical systems
GPS	Global position system
EDDIWPSO	Exponentially decreasing dynamic inertia weight particle swarm optimization
LDDIWPSO	Linear decreasing dynamic inertia weight particle swarm optimization
MSE	Mean square error
AHRS	Attitude reference system
